# Furuncular Cutaneous Myiasis in a Nontraveler: A Case Report

**DOI:** 10.7759/cureus.115181

**Published:** 2026-08-25

**Authors:** Hanan F Alotaibi, Samar Y Dilyh

**Affiliations:** 1 Family Medicine, King Abdulaziz Medical City, Jeddah, SAU

**Keywords:** cutaneous myiasis, fly larva, furuncle-like lesion, furuncular cutaneous myiasis, myiasis, parasitic skin infestation, saudi arabia

## Abstract

Furuncular cutaneous myiasis is an uncommon cause of a furuncle-like subcutaneous lesion and may be mistaken for a bacterial skin infection. We report the case of a previously healthy 30-year-old Saudi woman who presented with a one-week history of a progressively enlarging lesion on the posterior aspect of the left upper thigh. The lesion was initially asymptomatic but subsequently developed localized pruritus, intermittent sharp pain, and surrounding ecchymosis. One day before presentation, a live larva spontaneously emerged through a central punctum after the patient noted discharge from the lesion. She had no recent travel or recognized animal exposure but reported recent exposure to a recreational farm. Examination revealed a solitary subcutaneous lesion with a central punctum and minimal surrounding induration, without extensive erythema, fluctuance, or active discharge. The extracted larva was submitted to the parasitology laboratory for identification. The diagnosis of furuncular cutaneous myiasis was based on the characteristic furuncle-like lesion, central punctum, localized pruritus and pain, and spontaneous extrusion of a live larva. Following larval extrusion, the patient's pain resolved almost completely, with no further significant discharge reported. This case highlights the importance of considering furuncular cutaneous myiasis in the differential diagnosis of an atypical furuncle-like lesion, including in patients without a history of international travel.

## Introduction

Myiasis is a parasitic infestation caused by the larvae of dipterous flies that feed on living or necrotic tissue, body fluids, or ingested food [1,2]. Cutaneous myiasis is the most common clinical form and is broadly classified into furuncular, migratory, and wound myiasis [3]. Furuncular cutaneous myiasis is characterized by the development of a localized, boil-like subcutaneous nodule containing a single larva and typically presents with pruritus, pain, and a central punctum through which the larva obtains oxygen and intermittently drains serous or sanguineous fluid [2,3]. Although the condition is more frequently reported in tropical and subtropical regions and among international travelers, cases may also occur in individuals without recent international travel, depending on the distribution of the causative fly species and environmental exposure [3,4].

The clinical appearance of furuncular cutaneous myiasis may closely resemble common bacterial skin infections, particularly furuncles and cutaneous abscesses, which can result in delayed recognition and inappropriate treatment [1-4]. Diagnosis is primarily clinical and is supported by visualization or recovery of the larva. Complete removal of the larva is the cornerstone of treatment and is usually curative in uncomplicated cases [5-7]. This case highlights furuncular cutaneous myiasis in a patient without recent international travel or recognized animal exposure and emphasizes the importance of considering myiasis in the differential diagnosis of an atypical furuncle-like lesion.

## Case presentation

A 30-year-old previously healthy Saudi woman presented with a one-week history of a progressively enlarging swelling on the posterior aspect of the left upper thigh. The lesion was asymptomatic for the first two days, after which localized pruritus and intermittent sharp, electric shock-like pain developed. Over the following days, the lesion increased in size and became surrounded by ecchymosis. She had no fever, chills, constitutional symptoms, preceding trauma, or known insect bite.

One day before presentation, the patient noted a small amount of purulent-appearing discharge from the lesion. After cleansing the area with an alcohol swab, she observed the spontaneous emergence of a live larva through a central opening in the lesion. She preserved the larva in a sterile container and brought it to the clinic. Following extrusion of the larva, the pain resolved almost completely, and there was no further significant discharge.

The patient had no history of similar lesions and reported no recent travel, camping, hiking, livestock exposure, or direct contact with domestic or wild animals. She had, however, spent several days at a recreational farm approximately one week before symptom onset. Her medical and surgical histories were unremarkable, and she was not taking any regular medications.

On examination, she was afebrile and hemodynamically stable. A solitary subcutaneous lesion was present on the posterior left upper thigh, with a central punctum at the site of larval extrusion (Figure 1). There was minimal surrounding induration without fluctuance, extensive erythema, active drainage, or regional lymphadenopathy. The remainder of the examination was unremarkable.

**Figure 1:**
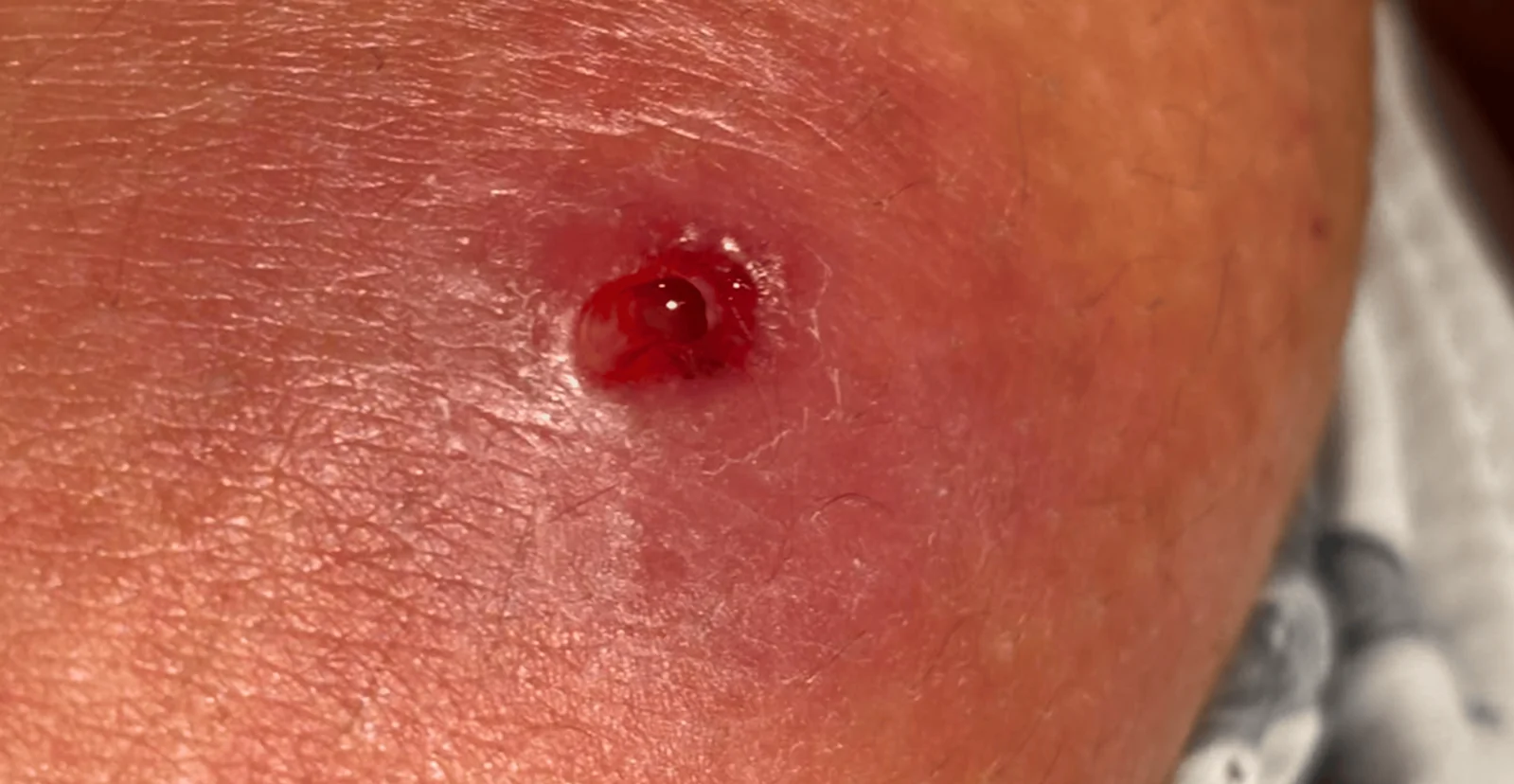
Extracted larva from the cutaneous lesion Clinical photograph showing a solitary furuncle-like subcutaneous lesion on the posterior aspect of the left thigh, with surrounding erythema and a central punctum through which the larva is visible at the time of spontaneous extrusion.

Clinical photograph showing a solitary furuncle-like subcutaneous lesion on the posterior aspect of the left thigh, with localized surrounding erythema and a central punctum at the site of larval extrusion.

The extracted larva was submitted alive to the parasitology laboratory for identification (Figure 2). The diagnosis of furuncular cutaneous myiasis was based on the characteristic furuncle-like subcutaneous lesion with a central punctum, localized pruritus and pain, and spontaneous extrusion of a live larva. The available clinical information does not establish the larva to species level.

**Figure 2:**
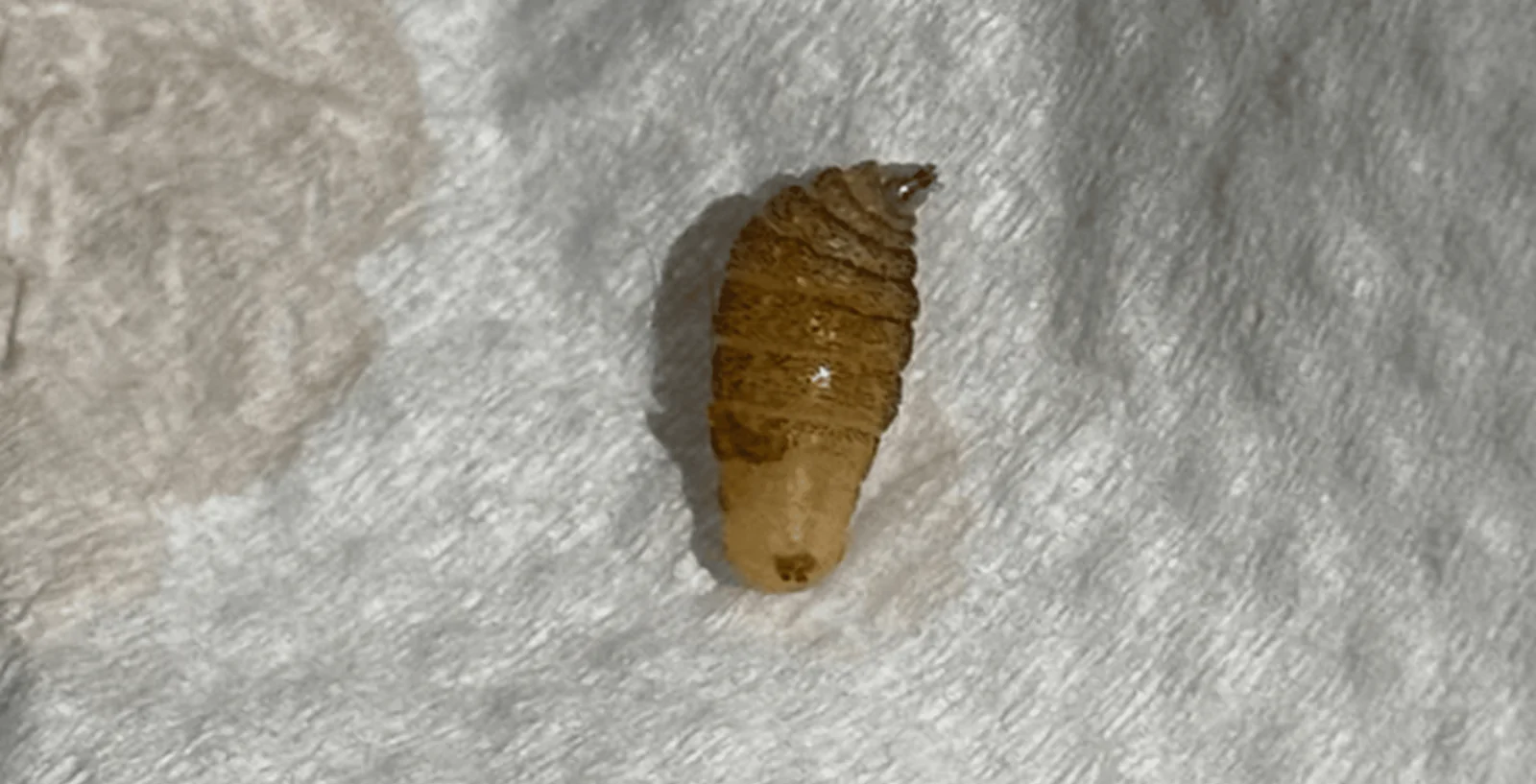
Extracted larva from the cutaneous lesion Live larva spontaneously expelled from the furuncle-like lesion and submitted to the parasitology laboratory for taxonomic identification

## Discussion

Furuncular cutaneous myiasis is an infestation of the skin by the larval stage of dipterous flies and is characterized by the development of a furuncle-like subcutaneous nodule containing a single larva [1,2]. Cutaneous myiasis is the most common clinical form of human myiasis and is classified into furuncular, migratory, and wound forms [3,5]. Furuncular cutaneous myiasis is most commonly associated with *Dermatobia hominis* and *Cordylobia* species, although other dipteran species may produce a similar clinical syndrome [4,5]. These species are discussed here as established causes of furuncular myiasis and should not be interpreted as identification of the larva in the present case.

The present case is notable because the patient had no history of international travel or recognized animal exposure. Although travel to endemic regions is an important diagnostic clue, its absence should not exclude furuncular cutaneous myiasis when the morphology and clinical evolution of the lesion are characteristic. The patient reported spending several days at a recreational farm approximately one week before symptom onset. This exposure was temporally associated with the development of the lesion; however, the available history does not establish the farm as the source of exposure or demonstrate the route by which exposure occurred.

The clinical evolution in this patient could initially have suggested a bacterial furuncle or cutaneous abscess. Furuncular cutaneous myiasis typically presents as a papule or nodule that progressively enlarges and develops a central punctum through which the larva obtains oxygen and releases fluid [1-4]. Pruritus and intermittent pain may occur, and the discharge may be serous, serosanguineous, or purulent in appearance, potentially contributing to diagnostic confusion with bacterial skin infection [3,6]. In this patient, the combination of a subcutaneous lesion, central punctum, localized pruritus, intermittent sharp pain, and spontaneous emergence of a live larva provided important clinical clues favoring myiasis.

The surrounding ecchymosis was an additional atypical feature. Various morphologic presentations of furuncular cutaneous myiasis have been described, including vesicular, bullous, pustular, erosive, and ulcerative lesions [3,6]. Therefore, the absence of a classic erythematous furuncle should not exclude the diagnosis. In the present case, the ecchymotic appearance contributed to the unusual presentation and further illustrates the potential for furuncular cutaneous myiasis to resemble other subcutaneous lesions.

Examination of an expelled or extracted larva can provide important diagnostic information. The diagnosis of myiasis is based on identification of a fly larva in affected tissue or examination of an extracted specimen. Species-level identification, when performed, may rely on morphologic characteristics such as the posterior and anterior spiracles, mouthparts, cephalopharyngeal skeleton, and cuticular structures, together with geographic and exposure histories [1-4]. In this case, the live larva was preserved and submitted to the parasitology laboratory for identification. However, the available case information does not provide the laboratory method, specific taxonomic result, or species-level identification. Accordingly, no specific fly species is assigned to this case.

Treatment of furuncular cutaneous myiasis is directed primarily toward complete removal of the larva [1,3,5]. In this patient, the larva had already spontaneously extruded before clinical presentation. Following extrusion, the patient's pain resolved almost completely, and no further significant discharge was reported. The available case information does not provide further details regarding the duration of follow-up, wound care, antimicrobial or antiparasitic treatment, or tetanus assessment and therefore does not permit conclusions regarding these aspects of management.

The clinical course immediately following larval extrusion supports the diagnosis and illustrates the improvement that may occur after removal of the larva. Secondary bacterial infection is a potential complication of furuncular cutaneous myiasis, although there were no clinical findings suggestive of secondary infection at the time of examination in this case.

This case emphasizes that furuncular cutaneous myiasis should be considered in the differential diagnosis of a persistent or atypical furuncle-like subcutaneous lesion, even in patients without recent international travel or recognized animal exposure. Recognition of a central punctum together with localized pruritus, intermittent pain, or a sensation of movement may raise suspicion for the diagnosis. When a larva is expelled or removed, preservation and examination of the specimen may provide additional diagnostic information and, when feasible, allow species-level identification.

## Conclusions

Furuncular cutaneous myiasis should be considered in the differential diagnosis of a persistent or atypical furuncle-like lesion, including in patients without a history of international travel or recognized animal exposure. In this patient, the combination of a central punctum, localized pruritus and pain, spontaneous extrusion of a live larva, and subsequent symptom improvement supported the diagnosis. Preservation and examination of an expelled specimen may provide additional diagnostic information and, when feasible, permit species-level identification. The recent recreational farm exposure was temporally associated with symptom onset, but the available information does not establish it as the source of exposure.
